# Investigating the human and nonobese diabetic mouse MHC class II immunopeptidome using protein language modeling

**DOI:** 10.1093/bioinformatics/btad469

**Published:** 2023-08-01

**Authors:** Philip Hartout, Bojana Počuča, Celia Méndez-García, Christian Schleberger

**Affiliations:** Discovery Sciences, Novartis Institutes for Biomedical Research, Basel 4056, Switzerland; NIBR Research Informatics, Novartis Institutes for Biomedical Research, Basel 4056, Switzerland; Discovery Sciences, Novartis Institutes for Biomedical Research, Basel 4056, Switzerland; Discovery Sciences, Novartis Institutes for Biomedical Research, Basel 4056, Switzerland

## Abstract

**Motivation:**

Identifying peptides associated with the major histocompability complex class II (MHCII) is a central task in the evaluation of the immunoregulatory function of therapeutics and drug prototypes. MHCII-peptide presentation prediction has multiple biopharmaceutical applications, including the safety assessment of biologics and engineered derivatives *in silico*, or the fast progression of antigen-specific immunomodulatory drug discovery programs in immune disease and cancer. This has resulted in the collection of large-scale datasets on adaptive immune receptor antigenic responses and MHC-associated peptide proteomics. In parallel, recent deep learning algorithmic advances in protein language modeling have shown potential in leveraging large collections of sequence data and improve MHC presentation prediction.

**Results:**

Here, we train a compact transformer model (AEGIS) on human and mouse MHCII immunopeptidome data, including a preclinical murine model, and evaluate its performance on the peptide presentation prediction task. We show that the transformer performs on par with existing deep learning algorithms and that combining datasets from multiple organisms increases model performance. We trained variants of the model with and without MHCII information. In both alternatives, the inclusion of peptides presented by the I-A^g7^ MHC class II molecule expressed by nonobese diabetic mice enabled for the first time the accurate *in silico* prediction of presented peptides in a preclinical type 1 diabetes model organism, which has promising therapeutic applications.

**Availability and implementation:**

The source code is available at https://github.com/Novartis/AEGIS.

## 1 Introduction

The major histocompatibility complex class II (MHCII) is expressed on the surface of professional antigen presenting cells (APCs). Its main function is the presentation of peptides from extracellular proteins to subpopulations of CD4^+^ T cells (Wieczorek *et al.* 2017). Co-stimulatory molecules expressed upon recognition influence CD4 ^+^ T cell activation and differentiation into either regulatory (T_reg_) or helper T cells (T_h_), which locally induce tolerance or promote inflammation (Mueller 2010, Pepper and Jenkins 2011, Graham and Xavier 2020, Murphy and Murphy 2022). Peptide presentation prediction by the MHCII has numerous biopharmaceutical use cases, such as enabling rapid progress in the development of immunomodulatory therapeutic strategies, or assisting in the selection of nonimmunogenic biologic drugs. Furthermore, computational selection of peptides for antigen-specific immune modulation deepens our mechanistic understanding in autoimmune disease and immune escape in cancer (Zhang *et al.* 2021).

The MHCII is a heterodimer containing an α and a β chain, encoded by the human leukocyte antigen (HLA) gene complex in humans and by the histocompatibility-2 (H-2) complex in mice. The structural features of the molecule influence peptide presentation. For instance, the binding groove ends of the MHCII are open, which allows for a higher diversity of ligands to be presented, compared to the closed-end groove of the MHC class I (Laimer and Lackner 2021, Wu *et al.* 2021). This promiscuity poses a major challenge for *in silico* techniques because long stretches of peptides do not contribute to the binding core, while the edges of the peptide could still contain information regarding the digestion process in the multivesicular antigen-processing intracellular compartments of the APC (Reinherz *et al.* 1999, Schneidman-Duhovny *et al.* 2018). Bioinformatic methods dedicated to the prediction of peptides presented by the MHCII need to take into account the binding core, the fraction of the peptide that interacts with the binding pocket of the MHCII molecule, and the amino acids that contribute the most to the binding core, referred to as anchor residues.

There are multiple strategies leveraging binding cores and anchor amino acids for the presentation prediction task, which is necessary for the classification of presented and nonpresented (or seldom-presented) peptides. These include position-specific scoring matrices (Nielsen *et al.* 2008), standard perceptron-based neural networks (Reynisson *et al.* 2020), convolutional neural networks (Racle *et al.* 2019), or transformers (Cheng *et al.* 2021). However, the variety of peptides presented poses significant challenges, lowering the performance of *in silico* methods. In this work, we aimed to leverage transformers due to their ability to capture long and varying ranges of dependencies in data, their computational efficiency, and their capability to handle input data of different dimensions.

## 2 Approach

We apply recent advances in natural language processing algorithms to peptides in order to investigate their ability to identify the MHCII immunopeptidome in humans and a preclinincal mouse model. We evaluated a transformer encoder on a predominantly human peptide:MHCII dataset and a second containing exclusively pancreatic islet MHCII peptidome data of nonobese diabetic (NOD) mice to assess predictive potential tailored to specific project needs. We validate the relative performance of our model against others published in the literature on the same data and determine that it outperforms or performs comparably to previously published strategies on the available feature sets.

## 3 Materials and methods

### 3.1 Datasets

To build the model, we sourced human (H) and mouse (M) peptide data from MHCII binding affinity (BA) and mass spectrometry (MS)-eluted ligand (EL) assays from the immune epitope database (IEDB) (Vita *et al.* 2019). The EL subset contains exclusively single-allele (SA) SEQ:MHCII information for nearly 1000 human (HLA-DR, HLA-DQ, and HLA-DP) and wild type mouse (H-2) alleles. These data were utilized together with multiallele (MA) information by Reynisson *et al.* (2020) to train NetMHCIIpan v4.0. The BA data includes SEQ:MHCII binding affinities retrieved from the IEDB and partially overlaps with the dataset used to generate NetMHCII v2.3 and NetMHCIIpan v3.2 by Jensen *et al.* (2018). A total of 447 232 peptides associated to 85 unique alleles were represented in the BA and EL IEDB-sourced datasets. These experimentally acquired (presented) peptides were distributed into folds as follows: 90%–95% of the data was assigned to the training set, 2.5%–5% to the validation set, and 2.5%–5% to the test set (Table 1).

**Table 1.** Descriptive statistics of the IEDB and NOD I-A^g7^ datasets used for training, validation, and evaluation of the model.^a^

Data source (organism)	Data type (*n* alleles)	Experimentally acquired			Synthetic		
		Train	Validation	Test	Train	Validation	Test
IEDB (H)	BA^b^ (*n* = 65)	97 223	4834	6109			
	EL (*n* = 19)	286 010	6965	7155	2 469 500	64 950	64 920
Total	77^c^	383 233	11 799	13 264	2 469 500	64 950	64 920
IEDB (M)	BA^b^ (*n* = 8)	2308	30	68			
	EL (*n* = 2)	35 085	695	750	385 195	10 055	10 055
Total	8^c^	37 393	725	818	385 195	10 055	10 055
NOD I-A^g7^ (M)	EL (*n* = 1)	4669	467	258	23 345	2335	1290

a IEDB contains both human (H) and mouse (M) peptides from BA and EL assays. The NOD I-A^g7^ repository consists of EL data only.

b No decoy BA data was generated (see Section 3.2 for further explanation). The distribution of the BA data labels can be found on Supplementary Fig. S1.

c Allele totals represent the total amount of unique alleles.

We set out to evaluate the usability of limited project-specific information by considering MHCII peptidome data from NOD mice, which express the I-A^g7^ variant of the MHCII molecule. We used the dataset generated by Wan *et al.* (2020), which includes peptides eluted from myeloid APCs contained in pancreatic islets, peripheral lymph nodes, and spleens of NOD mice. A total of 5394 EL-only data points were split into folds as described above (Table 1).

### 3.2 Negative data (decoys)

The nature of the EL data acquisition workflow does not allow for the identification of nonpresented peptides. These are required to train the binary machine learning classification model and were, therefore, generated *in silico*. The synthetic (negative data, decoys, nonpresented, rarely presented) peptides generated by Reynisson *et al.* (2020) were utilized. To create this set, peptides from the UniProt database were randomly and uniformly sampled for each length between 15 and 21 amino acids, including peptide flanking regions (PFRs) (UniProt Consortium 2021). A more specific approach was adopted for the decoys derived from NOD mice, where random peptides were sampled from nonpresented regions of proteins from which at least one peptide was presented. This ensured that relevant decoys were included in the model. For BA data, the experiment identifies rarely presented as well as nonpresented peptides. The affinity data points were log-transformed to a scale between 0 and 1 from experimental IC_50_ binding values as follows: 1–log(IC_50_ nM)/log (50 000) (Jensen *et al.* 2018, Nielsen *et al.* 2003) (Supplementary Fig. S1).

### 3.3 Data preparation

We relied on the method employed by NetMHCIIpan v3.0 to represent the MHCII binding pocket (Karosiene *et al.* 2013). In brief, a subset of amino acids was selected according to crystal structures of SEQ:MHCII complexes. The residues considered to be part of the binding pocket are located within 4.0 Å of the presented peptide. The resulting positions were used to build a pseudo sequence of the HLA, containing the most important amino acids determining the specificity of the binding pocket. It is worth noting that most of the polymorphism of the MHC regions occurred at those positions, further supporting the use of this feature compression method for the HLA. Following this method, we superposed the structure of I-A^g7^ (PDB-ID 1f3j) on the reference HLA-DR3 (PDB-ID 1a6a) and identified the residues in I-A^g7^ that spatially correspond to the HLA-DR3 pseudo sequence positions, as described in Karosiene *et al.* (2013). Thus, the extracted pseudo sequence representation corresponds with the reference representation (Supplementary Fig. S2). The relative mapping allows the combination of the IEDB and NOD mice datasets, considering the spareness of the latter.

The resulting feature set included the raw eluted peptide, ranging between 9 and 15 amino acids long, and the pseudo sequence. In addition, we built model variants without MHCII pseudo sequences to obtain a general presentability score regardless of MHCII allele.

The positional encoding is consistent with the original transformer paper from Vaswani *et al.* (2017). The maximum length of the input sequence is defined by the longest peptide sequence observed in the dataset (33), a start and stop token (2), and the length of the MHC pseudosequence (34). This parameter is modifiable, allowing the flexibility to modify the transformer’s code prior to commencing the training process. Its modification is contingent upon the set of features employed. Unused tokens are masked and handled in a manner akin to conventional sequence models. The embedding layer dimension is set to 128 for each residue.

An overview of the entire data processing pipeline can be found in Supplementary Fig. S3.

### 3.4 External datasets

We used independently acquired external databases to benchmark the performance of our model against published methods.

First, we focused on the data used to build the MARIA multimodal recurrent neural network (RNN) (Chen *et al.* 2019). These data include two ligandomes from K562 cells (leukemia-derived lymphoblasts) expressing the β-chain alleles HLA-DRB1^*^01:01 and HLA-DRB1*04:04, as well as an independent melanoma HLA-II ligand set. These data were chosen based on availability and comparability with the performance of different models. Unlike MARIA, our model does not integrate gene expression data, as this type of information is normally lacking. In addition, expression data can only be obtained for endogenous proteins or peptide tiles (if a library is being evaluated), therefore excluding exogenous antibodies, peptides, or engineered derivatives assayed *in vitro*. We have taken into account the recent report by Deng and Liu (2022), where a deep learning model for predicting MHCII antigen presentation is proposed. The performance of their model is compared with SOTA (state-of-the-art) methods on the two K562 cell line independent testing datasets described above.

In addition, we considered the HLA Ligand Atlas, utilized by Xu *et al.* (2022) as evaluation set for the FIONA-P model for MHCII surface presentation prediction. The Atlas consists of naturally eluted ligands from 29 tissues across 42 MHCII subtypes (Marcu *et al.* 2021).

### 3.5 Modeling setup

A variant of the model was trained excluding pseudo sequence information for both IEDB and the NOD mice dataset, which poses an advantage when allele-specific information is not available.

To divide our data into training, validation, and test sets, we randomly split the peptides and MHCII combinations across folds and subsequently isolated overlapping peptides to ensure the datasets were independent. The pan-specificity (i.e. ability to achieve high accuracy in the classification task for unseen alleles) of some predictors is often touted in the literature. We maintain that utilizing the model on novel alleles is not a predominant use case and that learning patterns of presented peptides related to known MHCII molecules is more useful and accurate.

**Figure 1.**
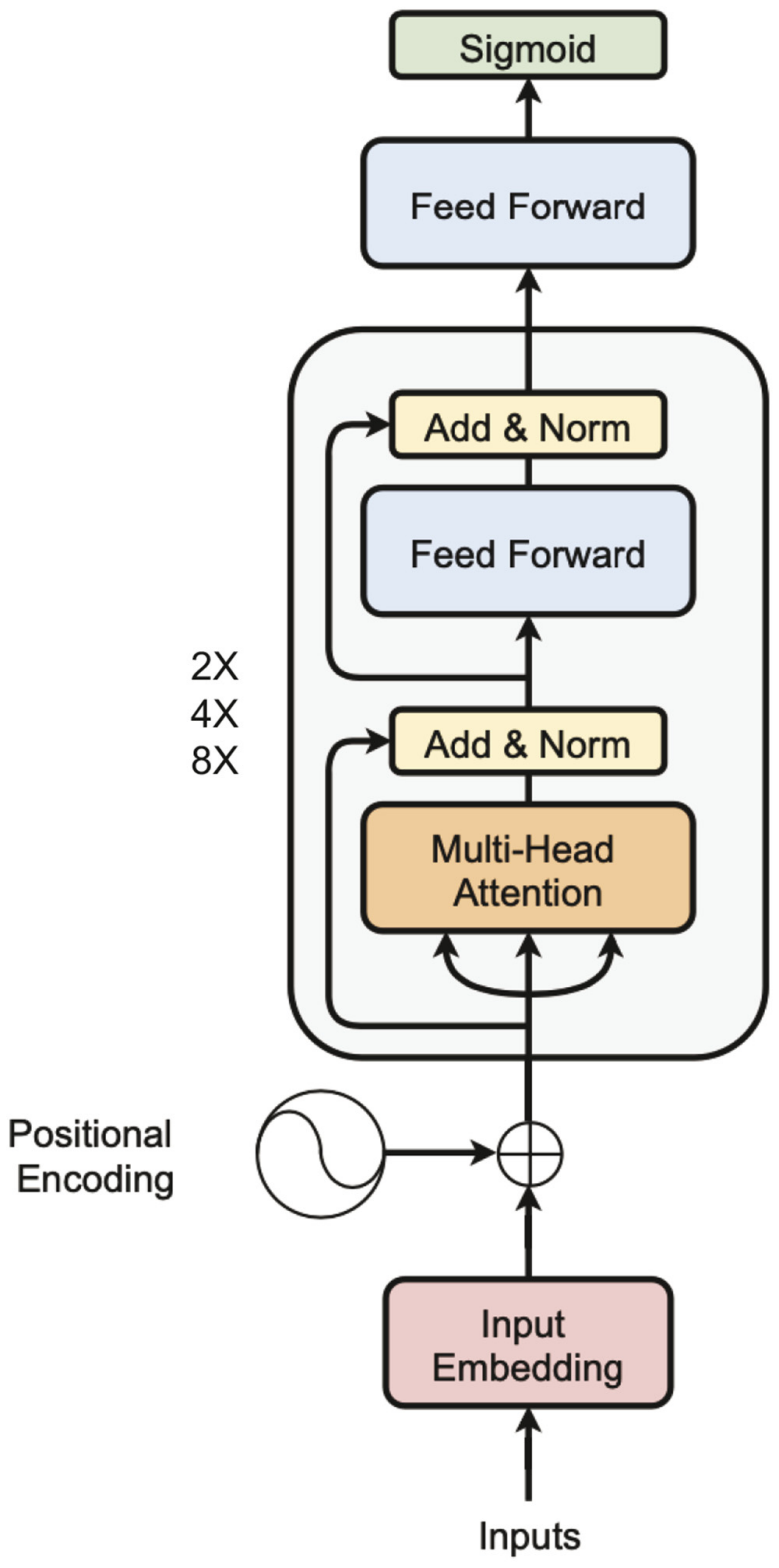
Transformer encoder architecture used to predict peptide presentation by MHCII; adapted from Vaswani *et al.* (2017). Instead of processing the data sequentially, the positional encoder embeds position-related information, which serves as input to a set of transformer blocks that extract features used for a small feedforward neural network, using these embeddings as features of prediction.

The architecture of the transformer model used in this study is shown in Fig. 1. In brief, the sequences are first marked with start and end of sequence tokens, then padded to ensure consistency of input to the model while avoiding spurious predictive power extracted from the padded characters. Then, the sequences are fed through an embedding layer and a positional encoder to ensure that the position of each amino acid relative to one another is encoded into the embedding. Subsequently, the sequence of (i) multi-head attention networks, (ii) an addition and normalization layer, (iii) a feed-forward network, and (iv) one last addition and normalization layer are added and repeated two, four, or eight times. Prior to computing the final sigmoid value used for classification, one last feedforward step processes the output of the aforementioned block. We used a variety of well-established metrics to assess the binary classification performance of machine learning models on imbalanced datasets, such as the receiver operating characteristic (ROC) curve, as implemented through scikit-learn’s metrics API (Pedregosa *et al.* 2011).

The transformer was trained using the binary cross-entropy loss function together with the Adam optimization algorithm for 300 epochs (Kingma and Ba 2014). The model hyperparameters can be found in the code, available at https://github.com/Novartis/AEGIS. In total, the transformer has approximately 3.5M trainable parameters. We trained all model variants using an Intel^®^ Xeon^®^ Platinum 8368 CPU @2.40GHz and 1 H100 GPU with 80GB of VRAM.

To assess the robustness of our model variations, we conducted training utilizing four different seed values. Furthermore, we strived to enhance the code’s portability by incorporating the pytorch-lightning framework, which significantly mitigates the need for redundant code and streamlines the training process of model variations. This is further facilitated by the integration of the hydra configuration framework.

## 4 Results

The sample size of each dataset is shown in Table 1. Table 2 contains the performance of the HLA-agnostic model (i.e. no pseudo sequence is given as input), referred to as SEQ, and the HLA-specific model, SEQ:MHC, both trained on IEDB, NOD, and IEDB + NOD data sources.

**Table 2.** Performance results averaged over four different seeds for each combination of dataset, feature set and number of layers.^a^

Data source	Features	Layers	Train			Validation			Test		
			AUC	F1	MCC	AUC	F1	MCC	AUC	F1	MCC
IEDB	SEQ:MHC	2	0.96±0.00	0.74±0.00	0.73±0.00	0.93±0.00	0.77±0.00	0.74±0.00	0.97±0.00	0.76±0.01	0.75±0.01
		4	0.97±0.00	0.77±0.01	0.75±0.01	0.93±0.00	0.77±0.01	0.75±0.01	0.97±0.00	0.77±0.02	0.75±0.02
		8	0.97±0.00	0.79±0.00	0.78±0.00	0.93±0.00	0.78±0.00	0.76±0.00	0.97±0.00	0.79±0.00	0.77±0.00
	SEQ	2	0.93±0.00	0.58±0.01	0.57±0.01	0.90±0.00	0.65±0.00	0.61±0.00	0.93±0.00	0.57±0.02	0.55±0.02
		4	0.94±0.00	0.63±0.01	0.62±0.01	0.91±0.00	0.67±0.01	0.64±0.01	0.94±0.00	0.61±0.01	0.58±0.01
		8	0.95±0.00	0.68±0.01	0.66±0.01	0.91±0.00	0.69±0.00	0.66±0.00	0.94±0.00	0.64±0.01	0.61±0.01
IEDB+NOD	SEQ:MHC	2	0.96±0.00	0.74±0.01	0.73±0.01	0.93±0.00	0.77±0.00	0.75±0.00	0.92±0.00	0.72±0.01	0.70±0.01
		4	0.97±0.00	0.76±0.01	0.75±0.01	0.93±0.00	0.77±0.00	0.75±0.00	0.92±0.00	0.74±0.01	0.72±0.01
		8	0.97±0.00	0.78±0.01	0.77±0.01	0.93±0.00	0.77±0.00	0.75±0.00	0.93±0.00	0.74±0.01	0.72±0.02
	SEQ	2	0.93±0.00	0.59±0.01	0.58±0.01	0.90±0.00	0.65±0.01	0.61±0.00	0.88±0.00	0.55±0.01	0.53±0.01
		4	0.94±0.00	0.63±0.01	0.62±0.01	0.91±0.00	0.68±0.01	0.64±0.01	0.89±0.00	0.58±0.01	0.56±0.01
		8	0.95±0.00	0.68±0.01	0.67±0.01	0.91±0.00	0.69±0.00	0.66±0.01	0.89±0.00	0.59±0.01	0.56±0.01
NOD	SEQ:MHC^b^	2	0.86±0.01	0.50±0.03	0.45±0.03	0.60±0.00	0.24±0.03	0.19±0.03	0.86±0.01	0.46±0.02	0.38±0.02
		4	0.85±0.00	0.44±0.01	0.40±0.01	0.60±0.00	0.24±0.02	0.17±0.02	0.86±0.00	0.46±0.04	0.37±0.03
		8	0.84±0.01	0.41±0.02	0.37±0.02	0.60±0.00	0.31±0.03	0.22±0.02	0.85±0.01	0.49±0.03	0.39±0.03
	SEQ	2	0.85±0.01	0.46±0.03	0.42±0.03	0.61±0.00	0.25±0.04	0.19±0.03	0.86±0.00	0.48±0.03	0.40±0.03
		4	0.84±0.01	0.45±0.04	0.40±0.03	0.61±0.00	0.26±0.03	0.20±0.02	0.85±0.01	0.45±0.05	0.37±0.06
		8	0.85±0.01	0.42±0.02	0.40±0.02	0.61±0.00	0.20±0.02	0.15±0.01	0.85±0.00	0.39±0.05	0.32±0.05

a The values in the table represent the results for each setting’s respective validation set. The model performance stability is shown by the standard deviation values. SEQ: peptide sequence.

b The pseudo sequence for this model is the same for all samples.

The best performing models displayed an AUC > 0.95 and corresponded to the IEDB and IEDB + NOD independent and identically distributed (i.i.d.) datasets including pseudo sequence information (SEQ:MHC). We observed that the 4-layer model delivers a notable improvement over the 2-layer model. However, increasing the layer count from four to eight does not yield significantly better results (Table 2). These models underperformed considering metrics with set thresholds, such as the F1, accuracy, precision, and recall. While performance is on average lower for the mouse data, possibly due to overfitting, it is still usable, especially in use cases where a model with high recall (specificity) is required.

The model displayed a median AUC > 0.90 for the most frequent alleles of the HLA-DR, HLA-DQ, and HLA-DP human isotypes (Greenbaum et al. 2011), with best values observed for HLA-DRB1*15:01 (AUC = 0.97), HLA-DRB1*16:01 (AUC = 0.97), HLA-DRB1*07:01 (AUC = 0.96), HLA-DRB1*01:01 (AUC = 0.96), HLA-DRB3*01:01 (AUC = 0.96), HLA-DRB1*04:05 (AUC = 0.95), HLA-DPA1*01:03/DPB1*06:01 (AUC = 0.95), HLA-DRB1*11:01 (AUC = 0.95), HLA-DRB1*03:01 (AUC = 0.95), HLA-DPA1*01:03/DPB1*02:01 (AUC = 0.94), HLA-DQA1*01:02/DQB1*06:02 (AUC = 0.93), HLA-DRB1*04:01 (AUC = 0.92), and HLA-DRB1*12:01 (AUC = 0.91). See Supplementary Tables S1–S4 for further details.

All four models were evaluated on the I-A^g7^ test set, to which the model had not been previously exposed (Table 3). The best performance (AUC = 0.88) was newly observed for the i.i.d. IEDB + NOD dataset containing pseudo sequence information.

**Table 3.** Inference results from the best performing model (NOD mouse-only model is excluded) evaluated on the test set that contains NOD mouse-only data (1630 peptides).^a^

Data source	Feature set	Metric		
		AUC	F1	MCC
IEDB	SEQ	0.72±0.01	0.18±0.05	0.12±0.05
	SEQ:MHC	0.71±0.03	0.12±0.06	0.08±0.07
IEDB+NOD	SEQ	0.78±0.01	0.27±0.05	0.23±0.05
	SEQ:MHC	0.88±0.01	0.61±0.02	0.57±0.03

a SEQ: peptide sequence.

The learning curves for each of the models can be found in Supplementary Fig. S4.

To interpret the results, we applied SHapley Additive exPlanations (SHAP) Lundberg and Lee (2017), as recently described for a long short-term memory network (LSTM) used for MHCI binding prediction by Dickinson and Meyer (2022). SHAP values are computed by considering all possible combinations of features and their contributions to the prediction. For example, for the MHCII task, SHAP can reveal how each amino acid contributes to peptide presentation prediction. Our SHAP analysis of predictions for four MHC:peptide complexes and the comparison with corresponding X-ray structures shows that the SHAP values reflecting the contributions of peptide amino acid positions correlate with the respective positions and interactions of these amino acids in the complex structures with having high values at so called anchor residues pointing deep into the binding groove and low values at residues pointing to the solvent (Supplementary Fig. S5).

### 4.1 Model performance on independent datasets

**Figure 2.**
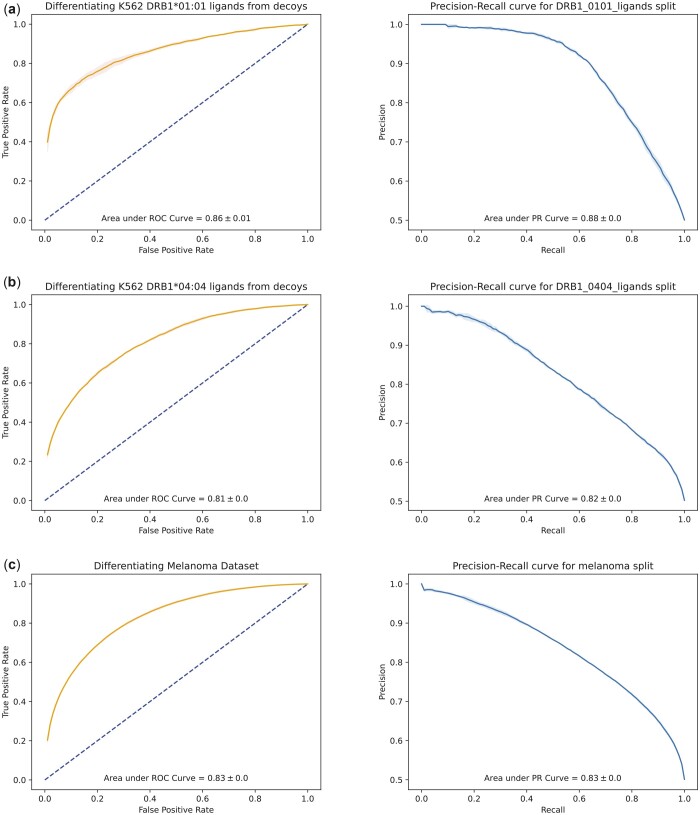
Receiver operating characteristic (ROC) and precision–recall curves of the transformer-based model on external datasets. Allele-specific model on the K652 cell-line data for the DRB1*01:01 (a) and DRB1*04:04 (b) alleles. (c) Performance of the allele-agnostic model on the melanoma dataset.

On all datasets, our model outperformed NetMHCIIpan v4.0 and v3.2 by a substantial margin (HLA-DRB1*01:01: our best model AUC = 0.86, NetMHCIIpan AUC = 0.608; HLA-DRB104:04: our best model AUC = 0.81, NetMHCIIpan AUC = 0.568). AEGIS underperforms compared to the MARIA multimodal RNN (HLA-DRB1*01:01: our best model AUC = 0.86, MARIA AUC = 0.885; HLA-DRB1*04:04: our best model AUC = 0.81, MARIA AUC = 0.892) (Fig. 2). Differently to AEGIS, MARIA utilizes gene expression data, which significantly contributes to the model’s performance; e.g. MARIA achieved an AUC = 0.81 in the classification task considering gene expression levels alone (Chen *et al.* 2019). Our approach also outperforms more recent models on the same MARIA data, with Deng and Liu (2022) achieving 0.71 and 0.84 AUROC on HLA-DRB1*01:01 and HLA-DRB1*04:04, respectively (Supplementary Table S5).

AEGIS marginally outperforms FIONA-P on the HLA Ligand Atlas (FIONA-P AUC = 0.91; our model AUC = 0.95) (Marcu *et al.* 2021, Xu *et al.* 2022).

## 5 Discussion

Overall, our results show that protein language models hold promise in the prediction of peptide presentation by the MHCII. Specifically on independently acquired experimental data, the model presented here performs similarly to models that leverage other features, such as gene expression. This is significant for biopharmaceutical settings where gene expression data cannot be acquired.

Our model performs comparably to BERTMHC, the first and most recent evaluation of a transformer neural network model on MHCII peptide data (Cheng *et al.* 2021). BERTMHC relies on the pretrained protein BERT (Bidirectional Encoder Representations from Transformers) model (Devlin *et al.* 2019). In brief, BERT is a bidirectionally trained language model that learns the context of a word based on its surroundings, therefore enabling the extraction of a deeper sense of context than single-direction models. Computationally, BERT consists of 12 layers with 12 attention heads per layer. In contrast, our model consists of four layers with two attention heads per layer. Both transformers are trained on IEDB-sourced immunopeptidome data, also utilized to train NetMHCIIpan versions 3.2 (BA information) and 4.0 (extending to EL data), with slight differences in data sizes [see Cheng *et al.* (2021) and Table 1 for details]. BERTMHC considers both multiallele (MA) and single-allele (SA) information, although a model trained in SA-only data (BERTMHC-SA) is also presented by Cheng *et al.* (2021). The performance of our model (SA data exclusively) is comparable to both BERTMHC and BERTMHC-SA, in spite of model size differences. In addition, both models are able to perform well (AUC > 0.75) on independent sets of data, consisting of binding affinity data from IEDB and patient-derived cancer immunopeptidome information in BERTMHC and two ligandomes from K562 cells as well as an independent melanoma MHCII ligand set reported by Chen *et al.* (2019) in our work.

We also describe an improved I-A^g7^-specific predictor which, despite leveraging a modest dataset for training, still performs satisfactorily on most metrics. The advantage of using such a transformer model on mouse data is that the training can be carried out in human, wild type, and NOD model mouse information simultaneously, hence applying patterns of immune recognition seen in other alleles and organisms while still identifying NOD mouse-specific signals. Thus far, the only available model for prediction of the I-A^g7^ immunopeptidome is PRED^NOD^, a scoring matrix-based method published by Rajapakse *et al.* (2006). The training and testing data of PRED^NOD^ were derived primarily from heterogeneous BA experiments, with a total of 653 experimentally acquired binders and 1000 nonbinders, whereas exclusively EL data (>20K peptides in the training set only) from a single experimental setup was utilized in our model. Our contribution offers a working alternative and extends the functionality of PRED^NOD^, suitable for the prediction of I-A^g7^ binders, rather than presented peptides.

While some important machine learning-related methodological advancements are presented here, there are still several sources of bias to consider and some shortcomings that need to be addressed in the future. First, to ensure feature consistency in our dataset, we omitted PFRs in our models. PFRs are the three residue positions before and after the peptide presented by the MHCII molecule on the source protein sequence. They have been reported to boost predictive performance and should be considered in future research (Barra *et al.* 2018).

It is also worth noting the limited difference in the model’s performance with and without inclusion of pseudo sequence information. In comparison with MHC class I data, where the incorporation of the MHC binding pocket pseudo sequences is significantly improving performance, MHCII data is relatively sparse, with a decrease of an order of magnitude compared to MHCI information (source IEDB) (Vita *et al.* 2019). This feature is likely to be driving the limited difference in performance between the SEQ and SEQ:MHC models.

There are also biases inherent to the data. We know that peptides with certain physico-chemical properties allowing for good detection are over-represented in mass spectrometry analytics (Jarnuczak *et al.* 2016), whereas e.g. cysteine-containing peptides are under-represented in MS-generated datasets (Abelin *et al.* 2017). In addition, BA data selects for peptides that bind to the MHCII molecule, exhibiting biochemical signature matches to MHCII molecules that may not be presented by the MHCII in nature, either because the peptide does not occur naturally or because it is being filtered by the complex intracellular dynamics involved in loading peptides onto the MHCII molecule (Karle 2020). The addition of these type of data to the training set may reduce performance in the *in silico* assessment of drug immunogenicity, while still producing usable results.

Furthermore, while sampling from UniProt to generate synthetic negative peptides as done by Reynisson *et al.* (2020) is an accepted practice across the literature to generate synthetic counterparts to the positive MS-data, it is not experimentally validated, and may produce sequences that could be in fact presented by the MHCII molecules.

Additional shortcomings are the inability of our method to handle multi-allele data for model variants requiring a pseudo sequence of the MHCII as input. This would require a semi-supervised learning setting, where peptides eluted from multi-allele cultures are assigned the most likely MHCII that presented each of them. This multi-label classification task could be enabled by a first classifier that learns to assign one or more MHCII molecules to each of the eluted peptides resolved using MS. Once the labeling of the multi-allele dataset is achieved, it could then be used as a larger pool of peptides for the main classifier predicting peptide presentation. This approach has led to one of the leading techniques for peptide presentation to leverage multi-allele data and thus, a wider coverage of many more real-life scenarios (Nielsen and Lund 2009, Reynisson *et al.* 2020). With this expanded dataset, more features could be added, such as information related to the peptide position in the protein structure and the source protein localization in the cell (Ten Broeke *et al.* 2013, Stern and Santambrogio 2016). In addition, building a differentiable encoding of the binding pocket from the entire MHCII pseudo sequence, possibly using protein language modeling, could also prove to extract more informative features than the currently used pseudo sequence.

Finally, while the randomly sampled peptides from UniProt contain peptides originating from multiple organisms, expanding the repertoire of presented peptides to bacterial species could further enhance the potential of our model to microbiome-related applications. This has been investigated before (Graham *et al.* 2018), but protein language models could further enhance the predictive performance of such models. In addition, we have shown that predictive performance in humans is sustained after the incorporation of MHCII equivalents from other organisms and that combining human data with information from different alleles and organisms might enable peptide presentation prediction by the MHCII molecule in species for which limited data are available.

## 6 Conclusion

Our work demonstrates that protein language models hold promise to tackle the difficult problem of identifying which peptides are presented by different MHCII alleles in homozygous data. Attention mechanisms are potent modeling strategies to identify short- and long-term dependencies between amino acids, which is ideal to extract features related to the PFRs, the binding core, and its interaction with the most important residues to determine the specificity of the open MHCII binding pocket. In addition, we have demonstrated the first protein language model specifically trained on I-A^g7^ data. As such, it can support efforts directed at leveraging NOD mice immunopeptidome measurements and drive therapeutic innovation.

## Supplementary Material

btad469_Supplementary_Data

## Data Availability

The data are archived permanently on Zenodo https://zenodo.org/record/8210867.
