# Supplementary material for: Investigating the human and nonobese diabetic mouse MHC class II immunopeptidome using protein language modeling
Source: Bioinformatics. 2023 Aug 1;39(8):btad469. doi: 10.1093/bioinformatics/btad469 (PMC10421966; doi:10.1093/bioinformatics/btad469)
Supplement: btad469_Supplementary_Data [file btad469_supplementary_data.pdf]

Supplementary Information

Supplementary tables

Supplementary Table 1: Model evaluation for an unbiased estimate of the model's performance in the most frequent HLA-DRB1 alleles.

Metric \ HLA-DRB1	01:01	03:01	04:01	04:05	07:01	08:02	09:01	11:01	12:01	15:01	16:01
**AUC**	0.96	0.95	0.92	0.95	0.96	0.72	0.77	0.95	0.91	0.97	0.97
**F1**	0.79	0.73	0.67	0.68	0.76	0.47	0.56	0.73	0.62	0.80	0.83
**MCC**	0.78	0.70	0.65	0.65	0.74	0.31	0.37	0.71	0.58	0.78	0.82

Supplementary Table 2: Model evaluation for an unbiased estimate of the model's performance in additional most frequent HLA-DR alleles.

Metric \ HLA-DR	B3*01:01	B3*02:02	B5*01:01
**AUC**	0.96	0.78	0.73
**F1**	0.80	0.51	0.49
**MCC**	0.77	0.44	28

Supplementary Table 3: Model evaluation for an unbiased estimate of the model's performance in the most frequent HLA-DQ allele pairs.

Metric \ HLA-DQ	A1*05:01 B1*02:01	A1*05:01 B1*03:01	A1*03:01 B1*03:02	A1*04:01 B1*04:02	A1*01:01 B1*05:01	A1*01:02 B1*06:02
**AUC**	0.71	0.73	0.70	0.64	0.73	0.93
**F1**	0.26	0.47	0.30	0.00	0.49	0.60
**MCC**	0.22	0.31	0.28	-0.07	0.45	0.59

Supplementary Table 4: Model evaluation for an unbiased estimate of the model's performance in the most frequent HLA-DP allele pairs.

Metric \ HLA-DP	A1*01:03 B1*02:01	A1*01:03 B1*06:01	A1*02:01 B1*05:01	A1*02:01 B1*14:01	A1*03:01 B1*04:02
**AUC**	0.94	0.95	0.80	0.73	0.78
**F1**	0.63	0.74	0.55	0.44	0.54
**MCC**	0.60	0.71	0.49	0.36	0.40

Supplementary Table 5: AEGIS performance compared to state-of-the-art methods. [(1)] Model reported in the current publication; [(2)] *Chen et al., 2019*; [(3)] *Andreatta et al., 2018*; [(4)] *You et al., 2022*; [(5)] *Deng & Liu, 2023*; [(6)] *Xu et al., 2022*; [(7)] *Chen et al., 2019*.

Model \ Dataset	**HLA Ligand Atlas** *Xu et al., 2022*	**K562 cell line DRB1*01:01** *Chen et al., 2019*	**K562 cell line DRB1*04:04** *Chen et al., 2019*	**Melanoma dataset** *Chen et al., 2019*
**AEGIS** [(1)]	0.95	0.86	0.81	0.83
**MARIA** [(2)]	0.87	0.89	0.89	0.89
**NetMHCIIpan 3.1** [(3)]	0.76	0.61	0.56	0.64
**DeepMHCII** [(4)]				
**Deng & Liu's model** [(5)]		0.71	0.84	
**FIONA-P** [(6)]	0.91			
**BERTMHC** [(7)]	0.89			

Supplementary figures

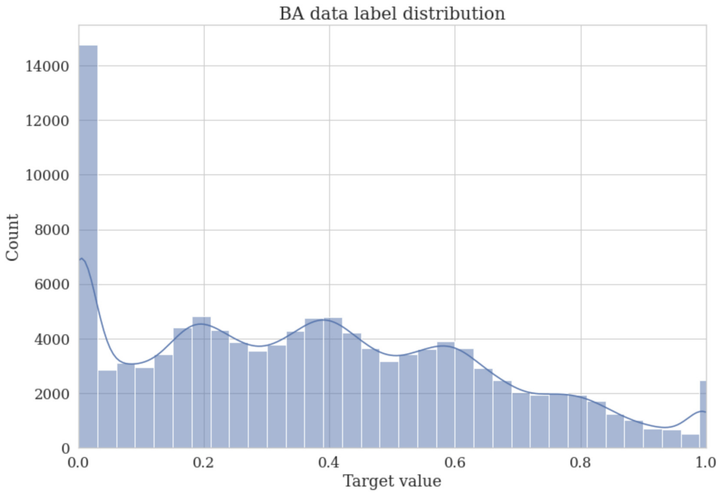

Supplementary Figure 1: Histogram of label distributions of the BA data

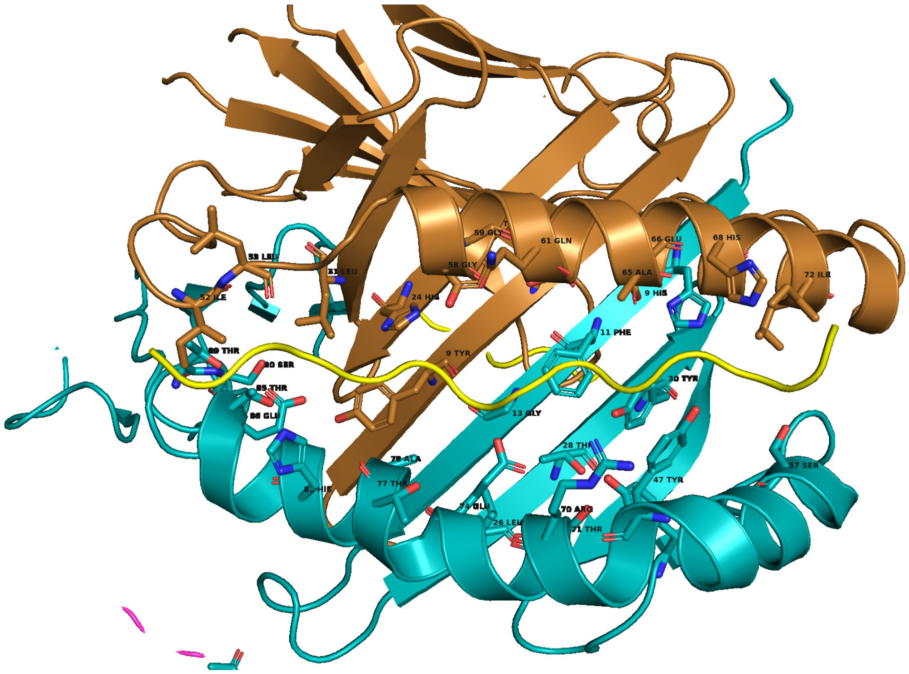

Supplementary Figure 2: Cartoon representation of the I-A^{g7} structure (PDB code 1f3j). The Aa chain is colored in copper, the Ab1 chain in teal, and the binding peptide in yellow. The residues conforming the pseudo sequence are indicated using a *ball and stick* representation and labeled.

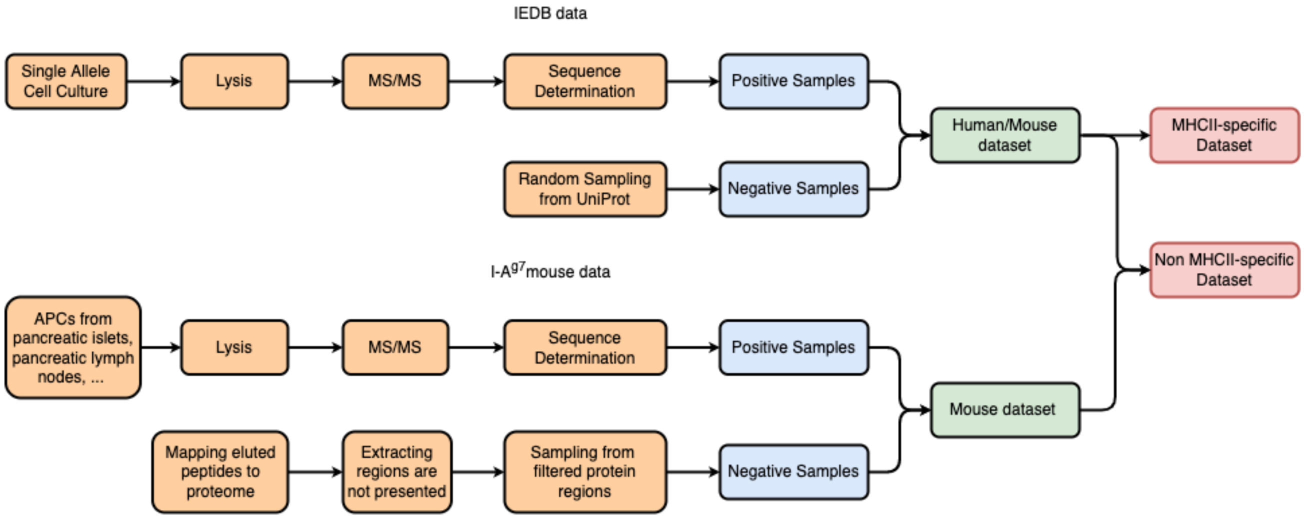

Supplementary Figure 3: Overview of the data processing and generation workflow.

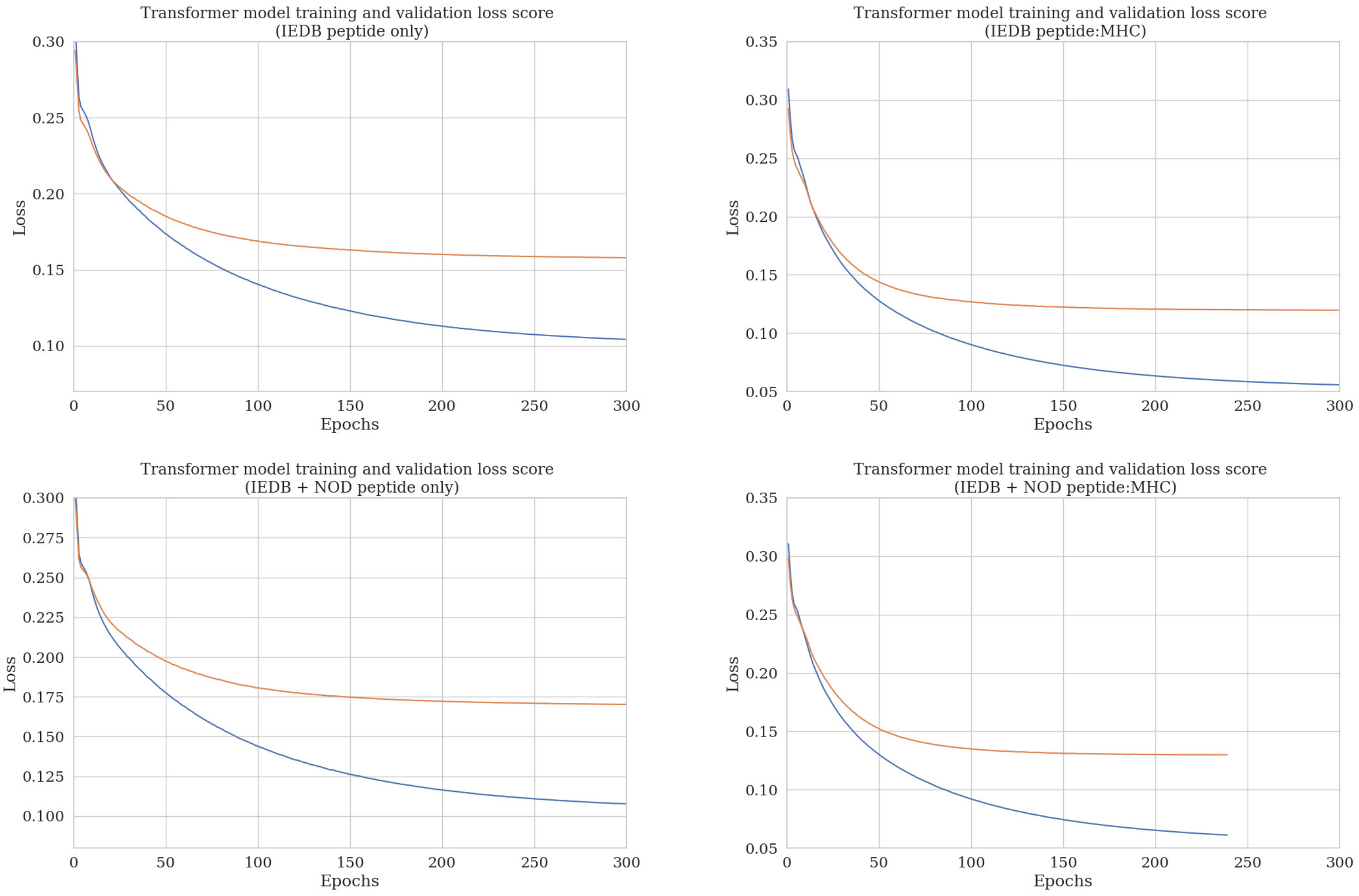

Supplementary Figure 4: Transformer learning curves with confidence intervals from four different training runs. Data sources and feature sets are indicated in brackets.

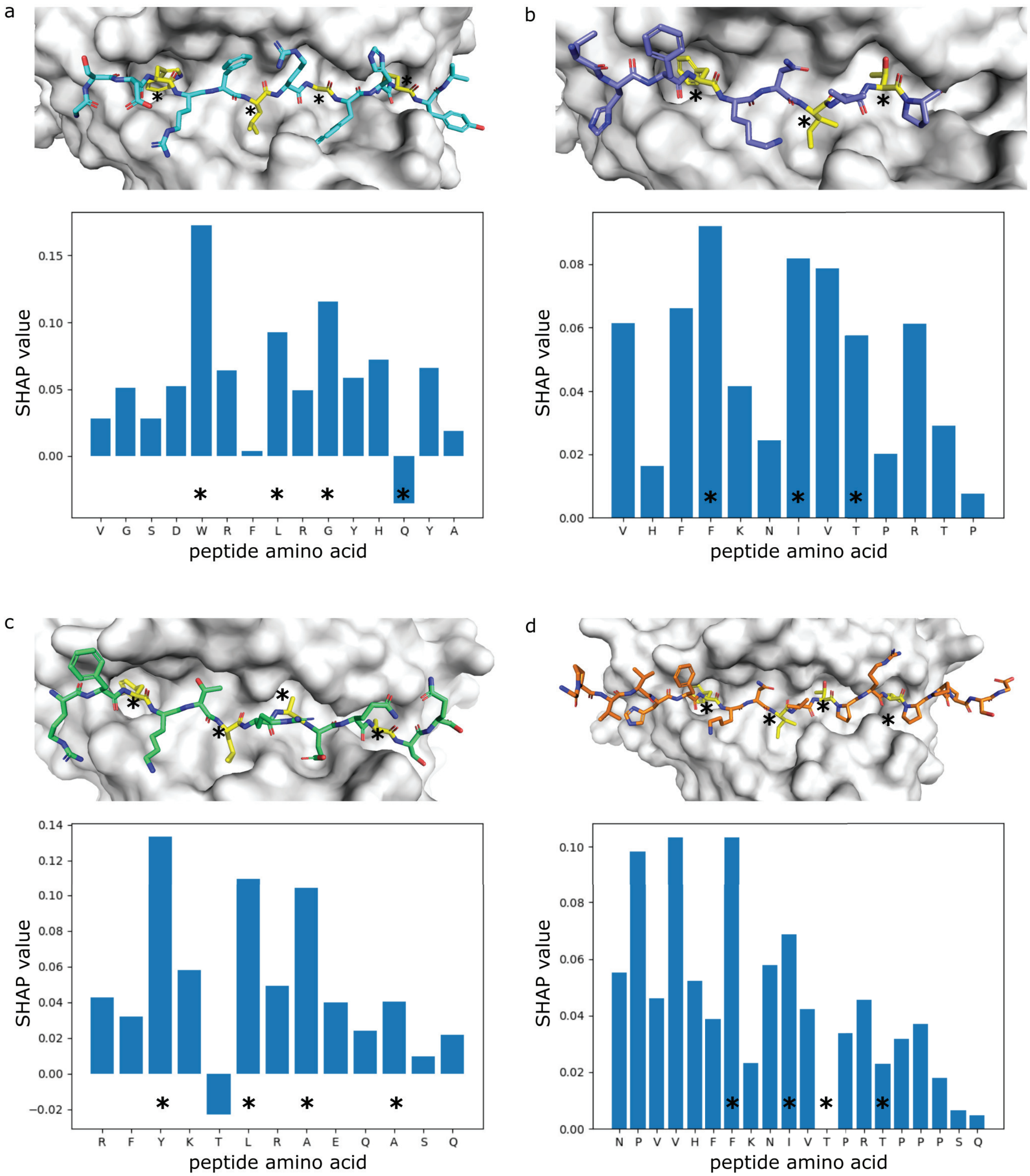

Supplementary Figure 5: SHAP analysis of predictions for four MHC:peptide complexes and the comparison with corresponding x-ray structures. The SHAP values reflecting the contributions of peptide amino acid positions correlate with the respective positions and interactions of these amino acids in the complex structures with having high values at so called anchor residues pointing deep into the binding groove and low values at residues pointing to the solvent.
